# 我如何诊断和治疗原发性血小板增多症

**DOI:** 10.3760/cma.j.issn.0253-2727.2023.01.005

**Published:** 2023-01

**Authors:** 磊 张, 荣凤 付

**Affiliations:** 1 中国医学科学院血液病医院（中国医学科学院血液学研究所），实验血液学国家重点实验室，国家血液系统疾病临床医学研究中心，细胞生态海河实验室，天津市血液病基因治疗研究重点实验室，中国医学科学院血液病基因治疗重点实验室，天津 300020 State Key Laboratory of Experimental Hematology, National Clinical Research Center for Blood Diseases, Haihe Laboratory of Cell Ecosystem, Institute of Hematology & Blood Diseases Hospital, Chinese Academy of Medical Sciences & Peking Union Medical College, Tianjin Key Laboratory of Gene Therapy for Blood Diseases, CAMS Key Laboratory of Gene Therapy for Blood Diseases, Tianjin 300020, China; 2 天津医学健康研究院，天津 301600 Tianjin Institutes of Health Science, Tianjin 301600, China

原发性血小板增多症（ET）是费城染色体阴性的慢性骨髓增殖性肿瘤（MPN）中较常见的亚型，年发病率为1～2.5/10万，发病高峰年龄在50～70岁[1]。ET起源于骨髓造血干/祖细胞的克隆性疾病，表现为巨核细胞过度增殖从而导致血小板计数明显增高[1]。ET的发病机制为基因突变或其他因素导致JAK-STAT信号通路高度活化[2]。ET的驱动基因突变包括JAK2 V617F、钙网蛋白基因（CALR）及骨髓增殖性白血病蛋白基因（MPL）突变，分别占50％～60％、15％～35％及2％～4％[3]–[4]。20％～30％的患者存在MPN非特异性基因突变（包括信号通路、转录因子、DNA甲基化、组蛋白甲基化以及剪接基因等）[5]。

一、典型病例

患者，女，28岁，因孕检发现“血小板增多4周”入院。入院4周前血常规：WBC 10.53×10^9^/L、RBC 4.6×10^12^/L、HGB 123 g/L、红细胞压积（HCT）36％、PLT 1180×10^9^/L、中性粒细胞绝对计数（ANC）7.4×10^9^/L，无不适症状。3 d前复查血常规：WBC 9.46×10^9^/L、RBC 4.8×10^12^/L、HGB 130 g/L、HCT 38％、PLT 1460×10^9^/L、ANC 6.9×10^9^/L，未治疗。既往体健，无吸烟、饮酒史，妊娠16周，孕1产0，否认家族类似病史及遗传病史。入院后血常规：WBC 9.56×10^9^/L、RBC 4.7×10^12^/L、HGB 125 g/L、HCT 37％、平均红细胞体积（MCV）85.7 fl、平均红细胞血红蛋白含量（MCH）28 pg、平均红细胞血红蛋白浓度（MCHC）327 g/L、PLT 1582×10^9^/L、ANC 7.1×10^9^/L；外周血乳酸脱氢酶（LDH）176 U/L（参考值0～248 U/L）；血管性血友病因子（VWF）抗原77％（参考值50％～160％），VWF活性58.1％（参考值48.8％～163.4％）；肝肾功能、电解质、铁代谢、C反应蛋白、凝血功能、肿瘤标志物、抗核抗体谱、狼疮抗凝物及抗磷脂抗体均未见异常。骨髓涂片：三系增生，骨髓及外周血血小板增多。骨髓病理：骨髓增生大致正常，巨核细胞显著增生，散在或簇状分布，巨核细胞胞体大、分叶多，网状纤维染色（MF-0级）。MPN基因突变筛查：CALR p.L367fs*46突变频率42％，BCR/ABL阴性。染色体核型为46，XX[20]。免疫分型：骨髓原始细胞比例不高，各系表型未见异常。B超：脾轻度增大。诊断：ET（修订版IPSET-thrombosis极低危组）、妊娠状态。给予聚乙二醇干扰素α-2b（PEG IFNα-2b）90 µg每周1次皮下注射，逐渐加量至180 µg每周1次，血小板计数降至1 000×10^9^/L以下后予阿司匹林100 mg每日1次，分娩前2周至分娩后6周改用低分子肝素，维持血小板计数（450～600）×10^9^/L。患者妊娠过程平稳，未发生血栓/出血事件；胎儿顺产，随访2年生长发育良好。

二、ET的临床表现

多数ET患者无不适症状，血栓/栓塞是最常见的并发症。诊断时或诊断前的血栓年发生率为10％～35％，诊断后的血栓年发生率为6％～10％[6]。动脉血栓更多见，而静脉血栓多发生于肝静脉、门静脉、脑静脉窦、肠系膜静脉等少见部位，布加综合征在年轻女性中发生率更高[7]–[8]。激光显微切割技术发现JAK2 V617F突变存在于肝、脾血管内皮细胞中，介导白细胞黏附、炎症反应等，从而导致肝、脾血管血栓形成[7],[9]。血栓的发生与JAK-STAT通路激活后产生的血小板本身功能及代谢异常、异常的免疫反应、血小板与血管内皮细胞之间以及血小板与其他血细胞之间的相互作用有关[10]。

3％～18％的ET患者有出血症状，严重出血的年发生率为0.79％[11]。获得性VWF多聚体缺乏是最常见的出血原因，VWF抗原和凝血因子Ⅷ检测无异常，而VWF功能评估（瑞斯托霉素辅因子活性）异常，血小板计数>1 000×10^9^/L时可能出现上述异常，血小板计数>1 500×10^9^/L时更加明显[3]。出血的其他原因包括获得性血小板储存池缺陷、肾上腺素能受体表达降低、对肾上腺素的反应受损、血小板表面糖蛋白受体表达下降等[3]。其他增加出血的危险因素还包括既往有严重出血史、白细胞计数显著增高、阿那格雷以及抗凝、抗血小板药物的使用等[3],[11]。

部分患者存在微循环障碍症状，包括头痛、头晕、晕厥、不典型的胸痛、肢体末端感觉异常、视觉异常、红斑性肢痛等。组织病理学显示红斑性肢痛部位存在富含血小板的小动脉微血栓伴随内皮炎症和内膜增生以及大量VWF沉积[3]。多数患者在使用抗血小板聚集或降细胞治疗后症状会得到缓解。

三、ET的诊断

建议采用2022年世界卫生组织（WHO）诊断标准[12]，此标准较2016年WHO标准[1]无变化。主要诊断指标：①血小板计数≥450×10^9^/L；②骨髓病理示巨核细胞高度增生，胞体大、核分叶过多的成熟巨核细胞数量增多，粒系及红系无显著增生或左移，且网状纤维极少轻度（1级）增多；③不能满足BCR-ABL阳性慢性髓性白血病、真性红细胞增多症（PV）、原发性骨髓纤维化（PMF）、骨髓增生异常综合征和其他髓系肿瘤的WHO诊断标准；④存在JAK2、CALR或MPL基因突变。次要诊断指标：存在其他克隆性证据或排除反应性血小板增多。符合4条主要标准或前3条主要标准和次要标准即可诊断ET[1],[12]。

诊断ET时应注意：①患者缺铁时要求补铁治疗不能使血红蛋白提高到PV水平。笔者曾多次见到由于铁缺乏以致HGB及HCT未达到PV诊断标准但最终诊断为PV的患者。②当血小板计数<450×10^9^/L但符合ET的其他诊断条件时，需关注患者是否存在由于ET或血栓因素导致脾脏显著增大而导致血小板破坏过多，同时也应首先排除PV（因脾大也会破坏红细胞）。③当患者红细胞表现为小细胞低色素、铁代谢指标不符合铁缺乏或补铁治疗效果欠佳时，应除外PV合并地中海贫血或其他遗传性溶血性疾病。④2016年WHO标准[1]、2022年WHO标准[12]以及新发布的国际共识[13]都强调了ET和原发性骨髓纤维化前期（Pre-PMF）的鉴别。在一项纳入1104例ET或Pre-PMF患者的研究中，Pre-PMF与ET的年死亡率分别为2.7％、1.3％，年白血病转化率分别为0.6％、0.1％，年显著PMF进展率分别为1％、0.5％，表明Pre-PMF的预后更差[14]。骨髓病理对于鉴别ET和Pre-PMF非常重要[15]。另外，Pre-PMF可能会有贫血、白细胞计数增高、可触及的脾大、乳酸脱氢酶升高、外周血出现幼红/幼粒细胞等表现[3]。⑤除了反应性血小板增多（包括感染、肿瘤、结缔组织病、铁缺乏、先天性脾缺如、脾切除术后、创伤、外科手术等），还应除外其他伴血小板增多的血液疾病（慢性髓性白血病、PMF、PV、慢性粒-单核细胞白血病、骨髓增生异常综合征/骨髓增殖性肿瘤伴环状铁粒幼红细胞和血小板增多、嗜酸性粒细胞增多症、骨髓增生异常综合征5q−综合征、淋巴细胞增殖性疾病等）。⑥对于年轻或儿童患者，应除外家族性或遗传性血小板增多症，涉及的突变位点包括THPO基因的5′-UTR或剪切识别位点、MPL S505N/P106L/W515R/K39N/R102P、JAK2 R564Q/V617I/R867Q等[16]。⑦对于反复发生罕见部位血栓形成、脾大伴血栓形成、脾切除后血小板持续增高的患者，应考虑是否有潜在ET可能，因ET表型可能在JAK2 V617F突变后多年才表现出来。

四、ET的预后判断

（一）总体生存预后判断

ET患者在诊断20年后的白血病转化率低于5％，骨髓纤维化进展率略高于白血病转化率。梅奥医学中心数据表明，ET患者的中位生存期约为20年，年龄小于60岁的患者为33年，但整体生存期短于年龄及性别匹配的正常人群[3],[17]。

2012年骨髓纤维化研究和治疗国际工作组提出了ET国际预后积分（IPSET）系统，依据年龄≥60岁（2分）、白细胞计数≥11×10^9^/L（1分）、有血栓史（1分），分为低危（0分）、中危（1～2分）和高危组（≥3分），三组的中位生存期分别为未达到、24.5年及13.8年[18]。随着二代测序技术的开展，研究发现基因突变也是影响生存的危险因素。一项纳入1607例ET患者的多中心回顾性研究发现JAK2 V617F突变负荷>35％、CALR Ⅰ型突变（或类似Ⅰ型突变）或MPL突变的患者进展为骨髓纤维化的风险更高[19]。近期还提出了突变驱动的国际预后积分（MIPSS）模型，根据高危基因突变（SRSF2突变、SF3B1突变、U2AF1突变或TP53突变）（2分）、年龄>60岁（4分）、男性（1分）、白细胞计数≥11×10^9^/L（1分），分为低危组（0～1分）、中危组（2～5分）、高危组（≥6分），各组中位生存期依次为34.4、14.1、7.9年[20]。

（二）血栓风险预后判断

动脉血栓的危险因素包括年龄>60岁、有心血管危险因素（CVF，包括糖尿病、高血压、高胆固醇血症或吸烟）、既往血栓史、JAK2 V617F突变及白细胞计数≥11×10^9^/L。血小板计数>1000×10^9^/L时，动脉血栓风险降低。静脉血栓危险因素包括男性、年龄>60岁、既往血栓史、JAK2 V617F突变阳性等[21]–[22]。2012年意大利学者提出了ET血栓国际预后积分系统（IPSET-thrombosis），依据有血栓史（2分）、JAK2 V617F突变（2分）、年龄>60岁（1分）、有CVF（1分），分为低危组（0～1分）、中危组（2分）、高危组（≥3分），三组血栓年发生率分别是1.03％、2.35％、3.56％[23]。后来Barbui等[22]提出了修订版IPSET-thrombosis，将ET分为极低危组（无血栓史、年龄≤60岁且JAK2 V617F突变阴性）、低危组（无血栓史、年龄≤60岁且JAK2 V617F突变阳性）、中危组（无血栓史、年龄>60岁且JAK2 V617F突变阴性）和高危组（有血栓史，或年龄>60岁且JAK2 V617F阳性），此模型已成为指导ET治疗的主要参考模型，笔者团队也证实了此模型同样适用于中国ET患者[24]。

五、ET的治疗

（一）一般治疗原则

目前ET的主要治疗目标为预防及治疗血栓并发症，可根据修订后的IPSET-thrombosis进行分层治疗[3],[25]–[26]。

1. 极低危组：如无CVF，观察随诊；如有CVF，予以阿司匹林100 mg每日1次。

2. 低危组：如无CVF，阿司匹林100 mg每日1次或每日2次；如有CVF，阿司匹林100 mg每日2次。

3. 中危组：降细胞治疗联合阿司匹林100 mg每日1次。

4. 高危组：年龄>60岁、无血栓病史且JAK2 V617F突变阳性者给予降细胞治疗联合阿司匹林100 mg每日2次。既往有动脉血栓病史者：①无CVF及JAK2 V617突变阴性者（任何年龄），予阿司匹林100 mg每日1次联合降细胞治疗；②年龄>60岁、有CVF或者JAK2 V617突变阳性者，予阿司匹林100 mg每日2次联合降细胞治疗。既往有静脉血栓病史者：①无CVF及JAK2 V617突变阴性者（任何年龄），给予降细胞联合系统性抗凝治疗；②有CVF或者JAK2 V617突变阳性者（任何年龄），给予阿司匹林100 mg每日1次联合降细胞及系统性抗凝治疗。

有任何以下特点的患者（危险分层不论）都需要考虑降细胞治疗[3],[25]–[26]：血小板计数>1500×10^9^/L，白细胞计数逐步增至>25×10^9^/L，脾肿大症状，严重的疾病相关症状。血小板计数>1000×10^9^/L或瑞斯托霉素辅因子活性<30％的患者慎用抗血小板聚集药物。有CVF者积极控制CVF。

（二）抗血小板或抗凝治疗

阿司匹林是最常用的抗血小板药物，对阿司匹林不耐受的患者可换用氯吡格雷或双嘧达莫。近期有研究表明，由于ET患者血小板代谢加快可能导致每日1次的阿司匹林剂量不足，每日2次的阿司匹林能更好地抑制环氧酶-1活性[27]，但是仍需要进一步研究评估不同剂量的阿司匹林在ET患者血栓预防中的疗效及安全性。目前正在进行一项Ⅱ期临床试验（AIRPORT-MPN），旨在比较直接口服抗凝剂阿哌沙班与阿司匹林在JAK2 V617F阳性MPN的初级血栓预防中的疗效和安全性，为采用直接口服抗凝剂作为ET的初级血栓预防提供依据。

（三）降细胞治疗

1. 一线药物

（1）羟基脲（HU）：HU是核苷酸还原酶抑制剂，在随机对照研究中被证明能降低高危ET血栓风险[3]。起始剂量为15～20 mg·kg^−1^·d^−1^，根据病情调整药物剂量。HU的并发症包括可逆的骨髓抑制和口腔黏膜或小腿溃疡形成，用于MPN是否有致白血病或第二肿瘤作用仍存在争议，因此一般在年龄较大的患者中使用。

（2）干扰素制剂：虽然没有直接证据可以证明重组干扰素α（IFNα）在预防血栓上的作用，但IFNα可以有效控制血小板数量，因其无致白血病或第二肿瘤作用，为年轻（年龄<60岁）患者的首选。PEG IFNα半衰期较IFNα长。相比于HU，IFNα不仅可获得相当的血液学缓解率，而且能降低JAK2 V617F或CALR的基因突变负荷、缩脾及缓解皮肤瘙痒[28]。MPN-RC 112随机对照临床试验比较了PEG IFNα-2a与HU在治疗高危ET和PV患者的疗效及安全性，治疗12个月后PEG IFNα-2a组与HU组在血液学缓解率方面相当，但治疗24～36个月时，PEG IFNα-2a组表现出更高的血液学及分子生物学缓解率，HU组则表现出更高的骨髓病理学缓解率，两组在血栓发生率及疾病进展率方面无差异[29]。JAK2 V617F的总体缓解率为30％～50％，完全缓解率为5％～10％，伴CALR突变患者的缓解率低于伴JAK2 V617F患者[29]–[30]，因此长期使用IFNα有改善病程进展的可能。这一理论引发了是否应将IFNα作为ET和PV的一线首选药物的思考，并对当前启动降细胞治疗的标准提出了挑战。

最近，一种新型长效单一异构体聚乙二醇化脯氨酸干扰素α-2b（ropeginterferon α-2b）在国外已获准用于治疗HU耐药/不耐受的PV患者，可每2～4周用药1次。一项全球多中心、随机对照的Ⅲ期临床试验（SURPASS-ET）正在进行，旨在比较ropeginterferon α-2b与阿那格雷治疗HU耐药或不耐受ET患者的疗效及安全性。Ropeginterferon α-2b用于治疗低危PV的多中心、随机对照Ⅱ期临床试验表明，标准治疗（阿司匹林及放血治疗）组疾病进展率为8％，而ropeginterferon α-2b联合阿司匹林及放血治疗组的疾病进展率为0[31]。此结果有望改变现有低危PV的治疗指南，但在低危ET中是否有同样疗效尚需证实。

重组IFNα起始剂量为3×10^6^ U每周3次皮下注射，起效后调整剂量。PEG IFNα起始剂量45 µg每周1次，可加量至180 µg每周1次，起效后调整剂量。IFNα最常见的不良反应为流感样症状、肝功能异常、甲状腺功能异常、自身免疫异常、抑郁等精神症状，治疗中需密切监测。

2．二线药物

（1）阿那格雷：是一种喹唑啉的衍生物，通过抑制巨核细胞分化降低血小板计数。起始剂量0.5 mg每日2次，最大单次剂量2.5 mg，最大每日剂量为10 mg，服用至少1周后再开始剂量调整，剂量增加每周不能超过0.5 mg/d。

两项随机对照研究比较了HU及阿那格雷在治疗ET中的疗效及安全性。其中一项纳入809例高危ET患者，HU组动脉血栓、严重出血及骨髓纤维化发生率均低于阿那格雷组，而阿那格雷组静脉血栓发生率较低，但阿那格雷组不良事件发生率较高[32]。另外一项研究纳入259例高危ET患者，两组的动脉/静脉血栓、出血事件、停药、血液学缓解及疾病进展发生率差异均无统计学意义[33]。另有研究指出，阿那格雷有增加骨髓纤维化转化或缩短总生存的风险[34]。阿那格雷可能发生进行性贫血、心悸、心律失常、体液潴留、心力衰竭或头痛等不良反应，慎用于老年和有心脏病史的患者。

（2）白消安、双溴丙哌嗪和^32^P：这些药物不除外导致白血病或第二肿瘤的风险，现已少用。

3. 靶向药物

芦可替尼（Ruxolitinib）是一种JAK1/2靶向抑制剂，主要用于中高危骨髓纤维化或HU耐药/不耐受的PV患者。在ET中，与常规治疗措施相比，芦可替尼治疗1年后的完全血液学缓解率、治疗2年后的血栓、出血发生率及疾病进展率差异均无统计学意义[35]。综合考虑到药物疗效、不良反应及经济因素，芦可替尼是否对ET患者有益尚需进一步研究。

4. 其他新型药物

赖氨酸特异性去甲基化酶1（LSD1）是一种表观调控酶，对恶性细胞的自我更新和造血分化至关重要。Bomedemstat（Img-7289）是一种口服LSD1抑制剂，2022年欧洲血液学年会更新了Bomedemstat作为二线药物治疗44例ET的结果：治疗12周后91％的患者血小板计数降至400×10^9^/L以下，治疗24周后83％的患者获得持久疗效，JAK2 V617F和CALR突变负荷也有不同程度的下降，69％的患者疾病相关症状得到改善。Bomedemstat的Ⅲ期临床试验正在计划进行。

端粒酶抑制剂Imetelstat作为二线药物治疗18例ET患者的Ⅱ期研究表明，所有患者均获得血液学缓解，8例JAK2 V617F突变的患者中7例获得分子生物学缓解，不良反应中肝功能异常比较常见，≥3级中性粒细胞减少的发生率为22％，贫血、头痛及晕厥的发生率为11％[36]。Imetelstat治疗ET表现出了较高的血液学及分子生物学缓解率，但其不良反应限制了该药的进一步推广。

（四）妊娠患者的治疗

ET患者妊娠早期的自然流产发生率>30％，显著高于正常人群（约15％），妊娠晚期产科并发症及母体严重血栓（发生率约5％）或严重出血事件（发生率约3％）比较少见，但也曾有报道指出ET患者较正常人群有更高的母体血栓形成和出血、先兆子痫、胎盘功能障碍、宫内生长受限、早产和死胎的风险[37]–[38]。血小板计数通常在妊娠中期和晚期显著下降，JAK2 V617F突变阳性是否影响患者的预后尚存在争议，IPSET-thrombosis高危组的流产率显著增高，阿司匹林及抗凝药物可以显著改善妊娠结局。虽然IFNα的使用不会增加胎儿或母体风险，但血小板计数和降细胞治疗似乎都不会影响产科并发症或妊娠结局，因此，不建议对低风险孕妇或备孕患者进行降细胞治疗[38]–[39]。

计划妊娠的患者，至少3个月前停用HU等可能致畸的药物。无妊娠合并症高危因素的患者，建议给予阿司匹林100 mg每日1次，分娩前2周至分娩后6周改用低分子肝素；有妊娠合并症高危因素者，给予阿司匹林100 mg每日1次联合低分子肝素至产后6周。妊娠合并症高危因素包括：血栓史，与ET相关的严重出血史，有可能存在由ET导致的妊娠合并症（不明原因的宫内胎儿发育迟缓、反复发生的非孕妇和胎盘因素妊娠10周内流产、妊娠>10周后正常发育胎儿的宫内死亡、因胎盘功能不全或严重的先兆子痫导致的妊娠<34周且正常发育胎儿的早产、严重的产前和产后出血、胎盘剥离等）。血小板计数>1500×10^9^/L时加用IFNα（建议首选醇化干扰素）[25]。阿司匹林会少量分泌入乳汁，低分子肝素不经乳汁分泌，二者均不影响哺乳。HU、IFNα等均会分泌入乳汁，因此用药期间禁止哺乳[40]。

本例患者血小板计数>450×10^9^/L，骨髓病理符合ET，CALR基因突变阳性，无其他髓系肿瘤表现，明确诊断为ET（修订版IPSET-thrombosis极低危组），因血小板计数>1500×10^9^/L，给予PEG IFNα-2b治疗，血小板计数低于1000×10^9^/L后给予阿司匹林100 mg每日1次，分娩前2周至分娩后6周改用低分子肝素，母体及胎儿均预后良好。
